# 3D-Printed Collagen-Based Waveform Microfibrous Scaffold for Periodontal Ligament Reconstruction

**DOI:** 10.3390/ijms22147725

**Published:** 2021-07-20

**Authors:** Hsu-Hsiang Lin, Pen-Hsiu Grace Chao, Wei-Chiu Tai, Po-Chun Chang

**Affiliations:** 1 Graduate Institute of Oral Biology, School of Dentistry, National Taiwan University, Taipei 10048, Taiwan; david905067@gmail.com; 2 Department of Biomedical Engineering, College of Medicine and College of Engineering, National Taiwan University, Taipei 10617, Taiwan; pgchao@ntu.edu.tw; 3 Graduate Institute of Clinical Dentistry, School of Dentistry, National Taiwan University, Taipei 10048, Taiwan; cpclab2014@gmail.com; 4 Division of Periodontics, Department of Dentistry, National Taiwan University Hospital, Taipei 10048, Taiwan; 5 School of Dentistry, College of Dental Medicine, Kaohsiung Medical University, Kaohsiung City 80708, Taiwan

**Keywords:** periodontal ligament, tissue engineering, bioprinting, collagen

## Abstract

Reconstruction of the periodontal ligament (PDL) to fulfill functional requirement remains a challenge. This study sought to develop a biomimetic microfibrous system capable of withstanding the functional load to assist PDL regeneration. Collagen-based straight and waveform microfibers to guide PDL cell growth were prepared using an extrusion-based bioprinter, and a laminar flow-based bioreactor was used to generate fluidic shear stress. PDL cells were seeded on the respective microfibers with 0 or 6 dynes/cm^2^ fluidic shear stress for 1–4 h. The viability, morphology, adhesion pattern, and gene expression levels of PDL cells were assessed. The results revealed that upon bioprinting optimization, collagen-based microfibers were successfully fabricated. The straight microfibers were 189.9 ± 11.44 μm wide and the waveform microfibers were 235.9 ± 11.22 μm wide. Under 6 dynes/cm^2^ shear stress, PDL cells were successfully seeded, and cytoskeleton expansion, adhesion, and viability were greater. Cyclin D, E-cadherin, and periostin were upregulated on the waveform microfibers. In conclusion, 3D-printed collagen-based waveform microfibers preserved PDL cell viability and exhibited an enhanced tendency to promote healing and regeneration under shear stress. This approach is promising for the development of a guiding scaffold for PDL regeneration.

## 1. Introduction

The periodontal ligament (PDL), a highly organized connective tissue structure situated between the alveolar bone and the teeth, plays important roles in transferring and dissipating loads from the occlusion [1]. However, periodontitis, a highly prevalent inflammation-induced destructive disease affecting up to 40–60% of people worldwide, frequently results in the damage or loss of PDL and leads to tooth hypermobility, reduction of the supporting bone, and even tooth loss [2]. The mechanical properties of PDL are largely determined by the orientation of collagen fiber bundles and the distribution of interstitial fluid [3]. The horizontal fibers are subjected to the greatest principal stress among the PDL fiber groups and exhibit the greatest strain under mastication [4]. Micromechanically, the collagen fibers are generally aligned according to a periodic crimped pattern [5]. When the ligament is stretched, the crimped fibers are straightened to bear the load and prevent overextension of the ligament [6,7]. Reconstructed PDL with an oriented fibrous microstructure similar to native PDL should be integral in the oral rehabilitation plan.

**Figure 1:**
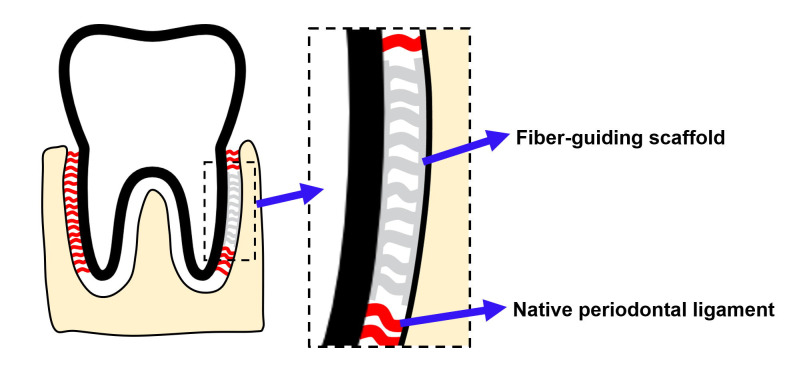
The concept of the fiber-guiding scaffold for PDL reconstruction. A fiber-guiding scaffold is composed of microfibers mimicking native PDL. When the scaffold is placed at the damaged PDL area, the microfibers will guide the growth of cells and formation of fiber bundles.

An ideal scaffold is crucial for regenerating PDL, and microfibrous scaffolds mimicking native PDL to guide PDL formation, so-called ‘fiber-guiding scaffolds’ (Figure 1), created by decellularizing tooth slices or 3D-printed (3DP) polymeric scaffolds, have been proposed as viable options to assist in the optimal reconstruction of PDL microarchitecture [8,9]. Decellularized tooth slices were reported to support the repopulation and differentiation of PDL cells [9]. However, the success of decellularization might be affected by the tissue conditions and decellularization protocols, and incomplete decellularization might induce an immune response that negatively influences the treatment outcome [10,11]. Polymeric 3DP scaffolds can produce intricate micro-patterned microarchitecture, which successfully aligns cells that guide fiber orientation in vivo [12]. However, one clinical case report revealed that a polycaprolactone-based polymeric scaffold did not satisfactorily regenerate the periodontium due to material-associated inflammation [13].

Collagen is the most abundant protein in the body and the major extracellular PDL protein. It has been widely used in dressings, hemostats, grafting materials, and scaffolds due to its excellent biocompatibility and weak antigenicity [14,15]. The major challenges in printing collagen include low viscosity, low denaturation temperature, and inferior mechanical properties [16,17]. A 3D printing technology termed freeform reversible embedding of suspended hydrogels (FRESH) was introduced recently and appeared to solve this dilemma [18]. Specifically, collagen was printed and deposited in a thermosensitive rigid hydrogel at room temperature. Once the entire 3D structure was accomplished, the hydrogel was melted and removed non-destructively. As reported by Lee and colleagues, FRESH technology enabled collagen printing to 20–200 μm resolution and full reproduction of the micro- and macrostructures of human hearts [19].

**Figure 2:**
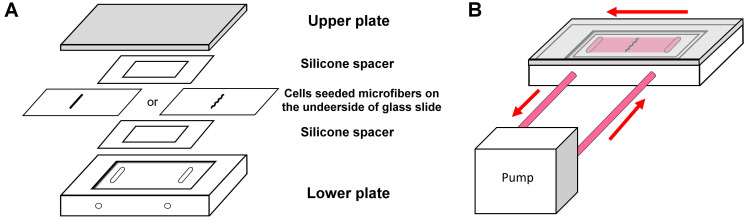
The designation of experiments. (**A**) The set-up of the parallel-plate flow chamber. (**B**) The set-up of the bioreactor.

This study aimed to develop a 3DP biomimetic fiber-guiding scaffold for PDL reconstruction. Collagen was chosen as the base material and scaffolds composed of patterned microfibers were prepared using FRESH technology. A laminar flow-based bioreactor, as shown in Figure 2, was used to simulate the loading condition of PDL, and the behaviors of PDL cells on the patterned microfibers under load were characterized in vitro.

## 2. Results

### 2.1. Optimization of Bioprinting for Collagen Microfibers

**Figure 3:**
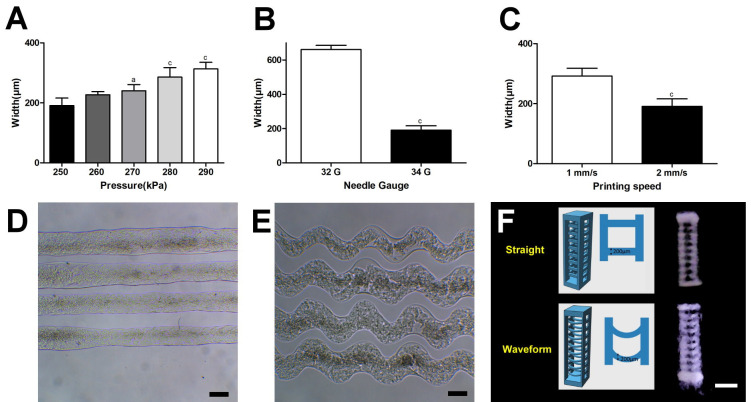
The characterization of the collagen microfibrous scaffold. (**A**–**C**) The optimization of the bioprinting process by printed line width analysis. (**A**) The printing pressure under the 34 G nozzle and the 2 mm/s speed (significant difference to 250 kPa printing pressure: a = *p* < 0.05, c = *p* < 0.001). (**B**) The nozzle diameter under the 250 kPa pressure and the 2 mm/s speed (significant difference: c = *p* < 0.001). (**C**) The printing speed under the 250 kPa pressure and the 34 G nozzle (significant difference: c = *p* < 0.001). (**D**,**E**) The resultant (**D**) straight and (**E**) waveform microfibers under the optimized condition. Scale bar: 200 μm. (**F**) The designed and resultant waveform microfibrous scaffolds for PDL regeneration. The dimensions of each microfibrous scaffold were designed to be 3 mm high, 1 mm wide, and 0.8 mm thick, and the thickness of the microfiber was designed to be 200 μm. Scale bar: 1 mm.

Higher printing pressure, wider nozzle diameter, and lower printing speed were associated with thicker microfibers (Figure 3A–C), and <250 KPa printing pressure or >2 mm/s printing speed caused difficulty in forming uniform lines (data not shown). Thus, the printing condition optimized for this study was 250 KPa printing pressure, 34 G nozzle, and 2 mm/s printing speed, and the resultant printed line width was 189.9 ± 11.44 μm for the straight microfibers and 235.9 ± 11.22 μm for the waveform microfibers (Figure 3D). The amplitude and wavelength of the waveform microfibers were 238.2 ± 17.12 μm and 750.2 ± 13.80 μm, respectively (Figure 3E). Figure 3F shows the cross-section of the resultant straight and waveform microfibrous scaffolds, which were, respectively, 3.19 ± 0.21 mm and 3.38 ± 0.28 mm high, and 0.85 ± 0.10 mm and 0.88 mm ± 0.08 mm thick. Due to the limited thickness of the scaffold, after subtracting the inner and outer walls, the width of the remaining space could only accommodate half a wavelength of the waveform microfiber in each scaffold.

### 2.2. The Morphology and Adhesion Patterns of PDL Cells

**Figure 4:**
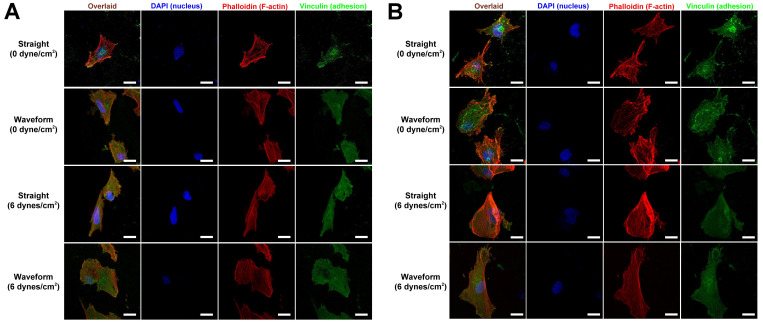
Morphology and adhesion patterns of PDL cells on the microfibers at (**A**) 1 h and (**B**) 4 h after initial seeding. Scale bar: 20 μm.

In general, PDL cells attached satisfactorily onto both straight and waveform microfibers and had spread more at 4 h. Compared with straight microfiber groups, cells spread more with less pronounced stress fibers in the waveform microfiber groups, especially under a shear load of 6 dyne/cm^2^ (Figure 4).

**Figure 5:**
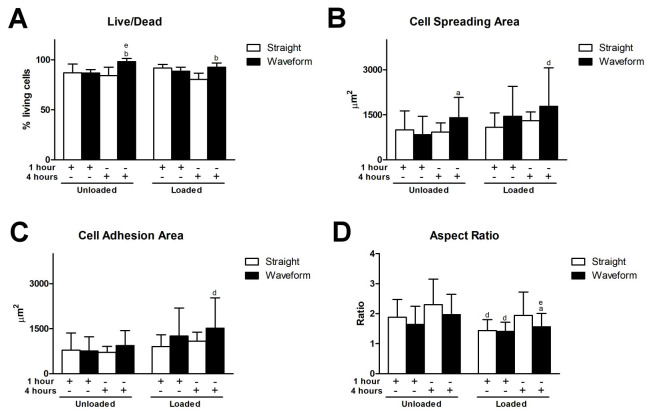
Behaviors of PDL cells on the microfibers. (**A**) The ratio of living cells. (**B**) Cell spreading area. (**C**) Cell adhesion area. (**D**) Aspect ratio. (Significant difference to the straight microfibers under the same loading condition at the same time point: a = *p* < 0.05, b = *p* < 0.01; significant difference to the straight microfibers without loading at the same time point: d = *p* < 0.05, e = *p* < 0.01).

The initial living cell density measured at 1 h was 899.28 ± 203.29 cell/mm^2^ on the straight microfibers and 983.90 ± 133.77 cell/mm^2^ on the waveform microfibers. Compared to the unloaded condition, cell spreading and adhesion areas were greater in those with a shear load (6 dyne/cm^2^). Compared to the unloaded straight microfiber group at 4 h, the cell spreading area was significantly greater in unloaded and loaded waveform microfiber groups (Figure 5A). Furthermore, the cell adhesion areas were significantly greater in the loaded waveform microfiber group (Figure 5B). The aspect ratio of the unloaded straight microfiber group was significantly greater than the loaded straight group at 1 h. It was also significantly greater than the loaded waveform microfiber group at 1 and 4 h (Figure 5C).

On the loaded straight microfibers, cell viability slightly decreased at 4 h. Therefore, compared with straight microfiber groups at 4 h, cell viability was significantly greater in both unloaded and loaded waveform microfiber groups (Figure 5D).

### 2.3. Gene Expression Profiles

**Figure 6:**
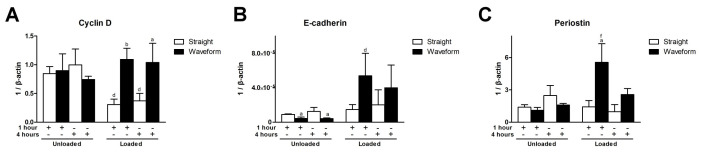
Gene expression profiles of PDL cells on the microfibers. (**A**) Cyclin D (*CCND1*). (**B**) E-cadherin (*CDH1*). (**C**) Periostin (*POSTN*). (Significant difference to the straight microfibers under the same loading condition at the same time point: a = *p* < 0.05, b = *p* < 0.01; significant difference to the straight microfibers without loading at the same time point: d = *p* < 0.05, f = *p* < 0.001).

Cyclin D is a regulator, controlling the G_1_/S phase transition in the cell cycle [20]. In the straight microfiber groups, cyclin D was downregulated under shear load at both time points. In the loaded condition, compared to the straight microfiber group, cyclin D was significantly upregulated in the waveform microfiber group at both time points (Figure 6A). E-cadherin, a mediator of cell-cell adhesion and cell-matrix interaction and a transmembrane mechanotransducer [21], was upregulated with shear load at both time points. In the unloaded condition, E-cadherin was significantly downregulated in the waveform microfiber groups relative to the straight microfiber groups. However, compared with the loaded straight microfiber group, E-cadherin was upregulated in the waveform microfiber group at both time points (Figure 6B). Periostin, a mediator involved in tissue healing and extracellular matrix assembly [22], was upregulated in the loaded waveform microfiber groups and significantly different from the straight microfiber group under the same loading condition at 1 h (Figure 6C).

## 3. Discussion

Although 3D printing technology appeared as an alternative to conventional scaffold fabrication, it is still challenging to print collagen due to the difficulty in preventing denaturation and scaffold collapse [19]. This study utilized FRESH technology to create a collagen-based microfiber scaffold, strengthened by light-activated riboflavin crosslinking [18,23]. Our optimization process revealed that the printing pressure, needle gauge width, and printing speed affected the printed line width and the printing quality. These results are consistent with a previous investigation [24]. Hence, the collagen slurry outputted by a 34 G needle gauge under a printing pressure of 250 kPa at 2 mm/s printing speed showed the most optimized printed line width (190.70  ±  25.33 μm), mimicking native PDL bundles, reportedly 100–200 μm thick [25].

As PDL consistently sustained loads from occlusion, this study utilized a laminar flow-based bioreactor to simulate the interfacial shear load on PDL. Shear stress had been considered the best approximation of biomechanical stress for studying PDL tissue engineering under physiological load [26]. Furthermore, under a shear stress of 3–15 dynes/cm^2^, PDL cells revealed morphologic changes, and F-actin rearrangement and signaling pathways involving extracellular remodeling and differentiation were upregulated [27,28,29,30]. In the present study, 6 dynes/cm^2^ of a shear load was utilized to prevent the disintegration or detachment of microfibers. Under shear load, cells were spreading with re-aligned F-actins, specifically at 4 h (Figure 4). As the cytoskeleton was linked with transmembrane molecules, the upregulation of E-cadherin, a transmembrane mechanotransducer, under loading (Figure 6B) indicates that a mechanical stimulus was transmitted to the cytoskeleton and promoted cell viability by increasing glucose uptake and ATP production [21,27]. The promotion of cell viability, in turn, reinforced the adhesion complex and cytoskeleton to resist external loads.

Waveform microfibers were fabricated to mimic the periodic crimped pattern of collagen fiber bundles in the ligament structure. This microcrimped morphology resulted in a bimodal stress-strain response, characterized by lower stiffness at low strain but increasing stiffness at higher strains, resulting in permanent deformation [31]. Cell morphology generally follows the microstructure of the substrate, known as contact guidance, and we found that PDL cells were spreading but were less aligned, with a lower aspect ratio, on waveform microfibers (Figure 4 and Figure 5). As Chao et al. indicated, on the microcrimped fibers, cells expressed more collagen and phenotypic markers, potentially through the interaction of Rho-associated protein kinase and a mammalian homolog of Drosophila diaphanous [32]. PDL cells were more viable and exhibited greater adhesion, spreading, and modeling potential on waveform microfibers under loading (Figure 4, Figure 5 and Figure 6). This phenomenon contributed to less deformation of waveform microfibers withstanding external loading. Furthermore, periostin, a mechanosensitive molecule essential for extracellular matrix assembly and tissue morphogenesis [22], was upregulated on waveform microfibers under loading (Figure 6C). Altogether, the research data supports waveform microfibers providing a favorable microenvironment to facilitate the growth and matrix synthesis of PDL cells in the presence of external loads.

The scaffold developed in the present study represents an attempt to promote PDL regeneration using aligned collagen microfibers capable of withstanding physiological loads. The scaffold architecture directed the growth of PDL cells and the fiber bundles toward the root surface, and hence mimicked the natural anatomy of PDL. Kim et al. reported that pre-loaded PDL cells on aligned fibrous scaffolds enabled more aligned PDL-like fibrous tissue formation in vivo [33]. However, the regeneration of the periodontal complex involves not only PDL but also mineralized structures, including alveolar bone and cementum, such that multiphasic scaffold designs may be required [34,35]. One concern in using the multiphasic scaffold was the weak interphasic cohesion, which affects the scaffold’s mechanical stability. Continuous additive manufacturing or simultaneous multiphasic cross-linking seem viable options to overcome this dilemma [34,36]. Furthermore, the implementation of tissue-specific bioactive molecules, such as enamel matrix derivatives, may further facilitate PDL formation and cementum of the scaffold-guided regeneration [37].

## 4. Materials and Methods

### 4.1. Preparation of 3D-Printed Collagen Microfibrous Scaffold

#### 4.1.1. Bioprinting Process

A highly concentrated type I collagen bioink (Lifelink^®^ 200, Advanced BioMatrix Inc., Carlsbad, CA, USA) was utilized to print the collagen microfibrous scaffold using the technique of freeform reversible embedding of suspended hydrogels (FRESH) [18]. In brief, a gelatin slurry support bath (GSSB) was formulated by mixing 150 mL 4.5% gelatin (ThermoFisher Scientific) with 350 mL 11 mM CaCl_2_ (Sigma-Aldrich, St. Louis, MI, USA) and was then centrifuged to remove the supernatant at 4200 rpm at 4 °C. The resultant GSSB was placed in a Petri dish prior to printing. The bioink was drawn into a 2.5 mL syringe and mounted into the syringe pump extruder on a bio-printer (INKREDIBLE, Cellink Inc., Gothenburg, Sweden). The tip of the syringe nozzle was positioned at the bottom of the GSSB. The optimal printing conditions were determined by applying a range of pressures from 200 to 300 kPa, nozzles from 28 G to 34 G, and printing speeds from 1 to 3 mm/s were used to print 200 μm-thick straight and waveform lines at room temperature. The printed constructs were cross-linked in 0.02% riboflavin under UV light irradiation for 30 min at a wavelength of 365 nm. They were then heated to 37 °C to melt the gelatin and rinsed with 1× PBS. The resultant printed lines were photographed using a digital imaging system (AxioCam ICc5, Carl Zeiss Microscopy GmbH, Munich, Germany) under a light microscope (Leica DM500, Leica Microsystems, Wetzlar, Germany), and the printed lines’ widths were measured using ImagePro Plus (Media Cybernetics, Rockville, MD, USA).

#### 4.1.2. Designation and Characterization of the Microfibrous Scaffold

The scaffold was designed using computer-aided design software (123D Design, Autodesk Inc., San Raphael, CA, USA). The dimensions were 3 mm high, 1 mm wide, and with a thickness of 0.8 mm. Straight or waveform microfibers that were 200 μm thick with a 250 μm interfiber distance were homogenously distributed and run cross-sectionally within the scaffold. These microfibers were connected by 200-μm thick microfibers on the surfaces of the scaffold. The designed model was converted to G-code using slicing software (Slic3r, Free Software Foundation, Boston, MA, USA) and was outputted using the FRESH technique with the optimized settings generated as presented in Section 4.1.1.

### 4.2. Cell Behaviors under Simulated Loads

#### 4.2.1. Bioreactor Set-Up

A modified parallel-plate flow chamber was used to introduce laminar flow shear stress to the cells cultured on the printed collagen construct. As shown in Figure 2A, the chamber consisted of a Plexiglas block with a glass window, a silastic spacer (McMaster-Carr, New Brunswick, NJ, USA), a glass slide with cell-seeded microfibers, another silastic spacer, and a covering block. These components were held together with screws, and laminar flows were infused into the chamber via a pair of salt tubes [38] (Figure 2B). The fluidic shear stress was generated by a pump based on the equation:

$$\tau =\frac{6\;\cdot \;\mu \;\cdot \;Q}{\omega \;\cdot \;h^{2}}$$

where *Q* refers to the flow rate, *τ* refers to the target shear stress on the cells, *μ* refers to the viscosity of the flow medium, *ω* refers to the width of the chamber, and *h* refers to the height of the chamber. To generate shear stress of 6 dyne/cm^2^, the flow rate generated by a motor was 4.2 mL/min.

#### 4.2.2. Viability, Morphology, and Adhesion Pattern of Cells

Immortalized human periodontal ligament cells (PDL cells; Applied Biological Materials Inc., Richmond, BC, Canada) were seeded on the 24-mm long collagen microfibers directly printed on the glass slide at a density of 4 × 10^3^ cells/fiber. After 2 h of initial seeding, the slide with microfibers was installed in the parallel flow chamber (Figure 1), and the viability and attachment of cells on both the straight and waveform microfibers were assessed at 1 and 4 h under 0 or 6 dynes/cm^2^ fluidic shear stress. The cell viability was evaluated using a live/dead assay (ThermoFisher Scientific Co., Waltham, MA, USA), and cell attachment was evaluated using a 4’,6-diamidino-2-phenylindole (DAPI) assay (to identify the cell nucleus; Sigma-Aldrich, St. Louis, MO, USA) with rhodamine-conjugated phalloidin (to identify the cell spreading area by staining with F-actin; Santa Cruz Biotech Inc., Dallas, TX, USA) and vinculin (to identify the cell adhesion area by staining the focal adhesion plaques; Abcam PLC, Cambridge, UK) following the manufacturers’ instructions. All the images were acquired using a confocal microscope (Carl Zeiss LSM880, ZEISS, Jena, Germany). The ratio of living cells was manually calculated at a magnification of 20X using ImageJ (NIH, Bethesda, MA, USA). With regard to the cell spreading area, cell adhesion area, and aspect ratio, only cells that did not exhibit cell-cell contact were included (>10 cells/group/time point) and the images were automatically thresholded and measured by the built-in functions of ImagePro Plus (Media Cybernetics, Rockville, MD, USA).

#### 4.2.3. Gene Expression Profiles

PDL cells were seeded on the 24-mm-long straight or waveform microfibers under a density of 4 × 10^6^ cells/fiber, and after the initial seeding of 2 h, cells underwent fluidic shear stress of 0–6 dynes/cm^2^. Cells were trypsinized after 1, 4, and 8 h, and mRNA was extracted using an RNA isolation kit (RNeasy Mini Kit, QIAGEN GmbH, Hilden, Germany). RNA was then reverse transcribed to cDNA, utilizing an iScript cDNA Synthesis Kit (Bio-Rad Laboratories Inc., Hercules, CA, USA). Gene expression was quantitatively analyzed with a real-time PCR system (ABI 7800, ThermoFisher Scientific Inc., Waltham, MA, USA) and sequence-specific TaqMan gene expression assays (ThermoFisher Scientific Inc., Waltham, MA, USA) for β-actin (housekeeping gene; *ACTB*; UniGene ID Hs.520640), cyclin D (*CCND1*; UniGene ID Hs.523852), E-cadherin (*CDH1*; UniGene ID Hs.461086), and periostin (*POSTN*; UniGene ID Hs.136348). The gene expression levels were normalized to β-actin.

### 4.3. Statistical Analysis

Statistical analysis was performed using GraphPad Prism^®^ (GraphPad Software Inc., San Diego, CA, USA). Unpaired *t*-tests were used to compare differences in gauge width, printing speed, and the difference between the straight and waveform microfibrous scaffold groups under the same loading condition at the same time point in vitro. One-way ANOVA followed by Tukey’s post hoc test was used to compare the differences among printing pressures and treatment strategies at the same time point in vitro. The data are expressed as means ± standard deviations and a *p*-value of less than 0.05 is considered statistically significant.

## 5. Conclusions

3DP collagen-based waveform microfibers mimicking PDL fibers withstood shear load, preserved PDL cell viability, exhibited an enhanced tendency to promote healing and regeneration, and could present a feasible approach to developing a fiber-guiding scaffold for PDL regeneration.

## Data Availability

The data that support the findings of this study are available from the corresponding author upon reasonable request.
